# Identification and Characterization of DM1 Patients by a New Diagnostic Certified Assay: Neuromuscular and Cardiac Assessments

**DOI:** 10.1155/2013/958510

**Published:** 2013-05-09

**Authors:** Rea Valaperta, Valeria Sansone, Fortunata Lombardi, Chiara Verdelli, Alessio Colombo, Massimiliano Valisi, Elisa Brigonzi, Elena Costa, Giovanni Meola

**Affiliations:** ^1^Research Laboratories-Molecular Biology, IRCCS Policlinico San Donato, Piazza E. Malan 2, San Donato Milanese, 20097 Milan, Italy; ^2^Department of Neurology, Stroke Unit and Centre for Neuromuscular Disease, IRCCS Policlinico San Donato, Milan, Italy; ^3^Service Lab, Fleming Research, Milan, Italy; ^4^Service of Laboratory Medicine, IRCCS Policlinico San Donato, Milan, Italy

## Abstract

The expansion of the specific trinucleotide sequence, [CTG], is the molecular pathological mechanism responsible for the clinical manifestations of DM1. Many studies have described different molecular genetic techniques to detect DM1, but as yet there is no data on the analytical performances of techniques used so far in this disease. We therefore developed and validated a molecular method, “*Myotonic Dystrophy SB kit,*” to better characterize our DM1 population. 113 patients were examined: 20 DM1-positive, 11 DM1/DM2-negative, and13 DM1-negative/DM2-positive, who had a previous molecular diagnosis, while 69 were new cases. This assay correctly identified 113/113 patients, and all were confirmed by different homemade assays. Comparative analysis revealed that the sensitivity and the specificity of the new kit were very high (>99%). Same results were obtained using several extraction procedures and different concentrations of DNA. The distribution of pathologic alleles showed a prevalence of the “classical” form, while of the 96 nonexpanded alleles 19 different allelic types were observed. Cardiac and neuromuscular parameters were used to clinically characterize our patients and support the new genetic analysis. Our findings suggest that this assay appears to be a very robust and reliable molecular test, showing high reproducibility and giving an unambiguous interpretation of results.

## 1. Introduction

In 1992, Myotonic Dystrophy type 1 (DM1) was shown to be caused by an expanded [CTG]*n* repeat in the 3′-untranslated region of the DMPK gene (dystrophia myotonica-protein kinase) in the chromosomal region 19q13.3 [1–3]. DM1 is the most common adult form of muscular dystrophy with a prevalence of 1 in 8000, characterized by progressive muscle weakness and atrophy, myotonia, early-onset cataracts and multiple organ involvement [4, 5]. The cardiac conduction system is selectively involved in DM1, and severe cardiac arrhythmias and respiratory insufficiency are the most frequent causes of death in these patients [6]. Currently, there are three known forms of DM1: “Mild”, “Classic”, and “Congenital.” The “Mild” form has its onset after 50 years, only manifesting cataracts, myotonia, and a mild degree of muscle weakness. “Classic” myotonic dystrophy has onset between 10 and 60 years, presenting with myotonia, muscle weakness, cataracts, smooth muscle and cardiac muscle involvement, and multiple organ involvement. “Congenital” myotonic dystrophy (CDM) is symptomatic at birth or within the first year of life, presenting with respiratory and feeding difficulties and severe developmental delay. This form is almost always maternally transmitted. The number of [CTG] repeats is highly polymorphic, in both healthy individuals and DM1 patients. According to the DNA testing guidelines of the EMQN (European Molecular Genetics Quality Network) [7], healthy individuals have alleles with between 5 and 37 [CTG] repeats [8–10], whereas in patients with clinical manifestations of DM1, the number of repeats varies from 51 to several thousands. Intermediate alleles with 38–50 triplets are not disease causing but they are considered as “pre-mutations”; repeats with alleles 51–100 are protomutations, both of which show increased instability towards expansions. Carriers of premutations or protomutations present no or few mild symptoms, such as cataracts [11, 12]. Anticipation is a specific event, where the number of repeats tends to increase as the disease is passed from one generation to the next, leading to increasing severity of symptoms and decreasing age of onset [13, 14]. Although there are studies demonstrating that the severity of phenotype, age of onset of myotonia and muscle wasting, and cardiac conduction abnormalities appear to be associated with an increase in the number of leukocyte [CTG] repeats [15–19], the relationship between phenotype and genotype is still controversial [20]. These conflicting results may be explained by the instability of [CTG] repeats and tissue specificity expression so that the expansion in leukocytes may underestimate the actual expansion in the specific tissues studied in the correlation analysis. 

Another feature of DM1 is the incomplete penetrance, characterized by variable clinical signs within individuals having the same expansion sizes or within families. Symptoms can be overlapping with other dominant noncoding expansion disorders, such as DM2 or spinocerebellar ataxias (SCAs) [21], and differential diagnosis based on clinical examination can be uncertain; therefore, genetic testing plays an important role in making an accurate diagnosis of DM1 disease because it allows direct detection of the [CTG] expansion. In fact, the direct DNA tests have reduced the number of invasive (muscle biopsy) and noninvasive, but relatively painful (electromyography) diagnostic techniques for the diagnosis of DM1 [22, 23]. The genetic tests are often used for symptomatic confirmatory diagnostic testing and predictive testing, after the finding of the mutation in an affected family member. It is also useful for prenatal diagnosis, in both amniotic fluid cells and chorionic villus samples (CVS) [24], for at-risk pregnancies after evidence of fetal hypotonia and reduced fetal movements, considering a possible maternal cell contamination. On one hand the testing is extremely helpful in identifying individuals who are asymptomatic or exhibit equivocal symptoms, such as cataracts. Many studies have described different molecular genetic techniques to detect DM1, but as yet there is no data on the sensitivity, specificity, and reproducibility of the techniques used so far in this disease. For this reason, in this work we developed a new molecular diagnostic assay, Myotonic Dystrophy SB kit, a standardized and certified method, based on the combination of Long-Polymerase Chain Reaction and Southern Blot Analysis (SBA), to better characterize the DM1 mutation in a cohort of clinically well-defined DM1 patients attending the Neuromuscular Clinic at IRCCS Policlinico San Donato. 

The principal aims of this study were to evaluate (i) the analytical performances of the Myotonic Dystrophy SB kit and (ii) the distribution of pathologic and normal [CTG] repeats in a population of northern Italy. 

## 2. Materials and Methods

Informed consent was obtained from all patients in our study. 

### 2.1. Subjects

From May 2010 to May 2012 a cohort of 113 patients attending the Neuromuscular Clinic at IRCCS Policlinico San Donato were subjected to the molecular genetics analysis for determination of DM1. All subjects were of Italian nationality and evenly distributed by sex and age: 39 female 34.5% (average age 40aa ± 19) and 74 male 65.5% (mean age 45aa ± 16). Patients were selected as follows: 20 DM1-positive (17.7%), 11 DM1/DM2-negative (9.7%), and 13 DM1-negative/DM2-positive (11.5%) from a previous molecular diagnosis with different “homemade” tests, including: Gold Standard Assay, TP-PCR, and Extra-Long-PCR with Southern Blot Analysis. The inclusion of patients with DM2 has allowed a better evaluation of the specificity of the new commercial kit. Sixty-nine subjects (61.1%) were new cases with unknown genotype but enrolled because they presented one or more of the following diagnostic criteria: positive family history, cataracts, myotonia, and proximal or distal weakness.

### 2.2. Muscle Strength Assessments

Muscle strength was evaluated using the modified Medical Research Council (MRC). 15 muscles on the left and right were included, adding up to a total of 150 for normal muscle strength. 

### 2.3. Cardiac Evaluation

All patients were subjected to standard ECG, 24-hour ECG-Holter monitoring, and 2D echocardiograms. 

### 2.4. DNA Isolation

The extraction of genomic DNA from peripheral blood in EDTA is that we performed with the commercial kit “High Pure PCR preparation kit Template" Roche. The quality and quantity of the extracted DNAs were determined by a spectrophotometer (NanoDrop). 

### 2.5. Components of the “Myotonic Dystrophy SB Kit” (Experteam s.r.l, Venezia, Italy)

Inside the kit there are a ready-to-use Master Mix (DM Master MIX), an Extra-Long Polymerase (DM DNA Polymerase), digoxigenin-labeled probe, DNA Molecular Weight Markers VII and VIII, DIG labeled (Roche Diagnostics), and step-by-step instructions and suggestions for optimization the analysis. 

### 2.6. Long-PCR and MethaPhore Analysis

one *μ*g of genomic DNA of each patient was amplified in a reaction volume of 100 *μ*L, containing 55 *μ*L of “DM Master MIX” and 4 *μ*L of “DM DNA Polymerase.” Forward primer was labeled at the 5′ end with fluorescent tag 6-FAM. PCR conditions were one cycle of 1 min at 94°C; 28 cycles of 20 sec at 94°C and 7 min at 62°C; and finally 10 min at 72°C. The amplifications were performed by MyCycler instrument (BioRad). After the amplification 20 *μ*L of each PCR product was run on 3.5% MethaPhore agarose gel at 100 V and stained with ethidium bromide. Alleles with less than 100 repeats were analyzed by capillary electrophoresis on 3500 Genetic Analyzer (Applied Biosystems) using LIZ600 as size standard. The analysis of results was performed using GeneMapper v4.1 (Applied Biosystems). For alleles with more than 100 repeats Southern Blot hybridization was performed using the DIG-labeled probe as described in the next section.

### 2.7. Southern Blot Analysis

Thirty-five microliters of PCR products were separated by electrophoresis on 1% agarose gels, transferred to Nylon Membranes (Roche Diagnostics) and hybridized overnight with a nonradioactive Digoxigenin-based probe, 5′DIG-labeled [CTG]_10_. After being washed, the blots were incubated with antidigoxigenin alkaline phosphatase conjugate (AP) (Roche Diagnostics), and this one was detected by the addition of ready-to-use CDP Star (Roche Diagnostics). The chemiluminescence signal was visualized on the ChemiDoc Instrument (BioRad) after several exposures. finally we compared the bands obtained with two DNA molecular weight markers, DIG labeled.

### 2.8. Molecular Diagnosis

The genetic diagnosis was based on the guidelines of the EMQN; the technical validation of each analytical run was subjected to internal quality controls evaluation. Positive (high and low) and negative controls were patients with a previous molecular diagnosis. In addition after the genetic test, each patient was retrospectively reviewed for the distribution of pathologic and normal alleles, containing [CTG] repeats. The [CTG] repeats size in each allele was determined by capillary electrophoresis or by Southern Blot Analysis.

### 2.9. TP-PCR

TP-PCR was performed as discussed elsewhere [25, 26], using 500 ng of genomic DNA, after the amplicons were analyzed by capillary electrophoresis.

### 2.10. Statistical Analysis

The [CTG] expansion of each group was expressed as mean ± standard deviation and range. Statistical analyses were evaluated by Student's *t*-test. Probability values *P* < 0.05 were considered statistically significant.

## 3. Results

### 3.1. Assay Performance Characteristics

**Figure 1:**
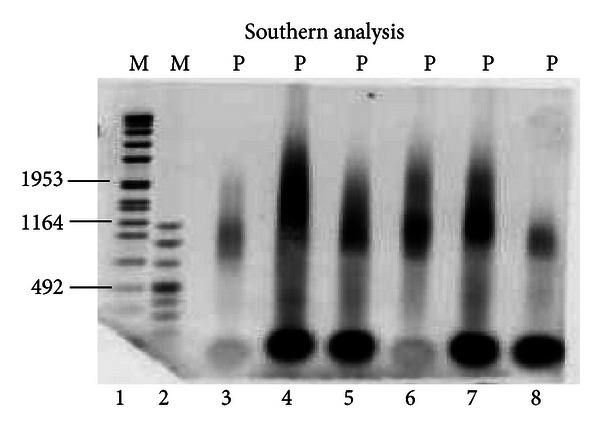
Expansion detection by Southern Blot Analysis. Lanes 1 and 2 are DNA molecular weight marker, (VII and VIII, resp.; Roche Diagnostics). Results for DM1 affected individuals are shown in lanes 3 to 8.

113 patients (74 males and 39 females) were subjected to molecular analysis using the new commercial assay Myotonic Dystrophy SB kit, for detection of DM1 disease, including those in whom genetic test results were previously known from other laboratories using previous nonstandardized techniques. Out of 113 individuals, sixty-five patients (57.5%) were diagnosed genetically as DM1 patients and forty-eight (42.5%) as DM1-negative patients. The assay confirmed the diagnosis in the 20 patients in whom previous testing had shown [CTG] expansions consistent with DM1 and it also confirmed normal [CTG] expansions in the 11 patients in whom DM1 or DM2 had been previously ruled out. All patients with the genetic diagnosis of DM1 fulfilled clinical and laboratory criteria for the disease. None of the 13 patients with DM2, who had been included in this study to increase the specificity, had [CTG] expansion size consistent with DM1. Sixty-nine new cases, with unknown genotype, were correctly identified by new molecular assay and divided as follows: forty-five were DM1-positive and twenty-four were DM1-negative. Results of the new molecular assay were compared with “homemade assays” on 113 DNA samples (Table 1). Comparative analysis revealed that the sensitivity, the specificity, and the accuracy of Myotonic Dystrophy SB kit were very high (>99%). No false-negative results or failed amplifications were observed. Furthermore, we obtained the same results using several procedures for extraction of genomic DNA, from fresh or frozen blood samples, with different concentrations of the same (300–1000 ng of genomic DNA). In addition, we checked interrater reliability by checking results from three different lab technicians, and we found that diagnostic performances of different operators with different degrees of experience were similar. Another feature of this molecular diagnostic test was the best resolution of larger expansions, which appear as single and well-defined bands especially if we consider that expanded alleles often appear as a smeared signal due to the somatic instability of the mutation (Figure 1). 

### 3.2. Pathologic [CTG] Distribution

We investigated the distribution of [CTG] repeats in all 113 individuals. In our cohort of 65 of 113 affected patients, the expanded alleles ranged from 70 to 2500 [CTG] repeats (mean ± SD size of the [CTG] repeat expansion; 385 ± 396 repeats, range 70–2500). According to EMQN 2008 classification for DM1 (Table 2), DM1 is classified into premutation (range 38–50 [CTG]), “Mild” form (range 51–149 [CTG]), “Classic” form (range 150–2000 [CTG]), and “Congenital” form (range > 2001 [CTG]). Because the range of [CTG] repeats in the “Classic” form is very large and may include patients with borderline expansions in the mild range and patients with expansions close to those in the “congenital” range, in this work we separated this range in two specific classes: *E2a* class ranging from 150 to 450 [CTG] and *E2b* class ranging from 451 to 2000 [CTG]. The majority of pathologic alleles, about 83.1% (54/65), were in the range of “Classic” phenotype. In particular 42/65 patients (64.6%) showed *E2a* -genotype and 12/65 patients (18.5%) presented *E2b* -genotype. The remaining 13.9% (9/65) had the “Mild” form, between 51 and 149 repeats. Only two cases were found with the [CTG] expansions over 2000 repeats (3.1%). The verification of the expanded size was obtained by automated capillary electrophoresis, because the amplicons were labeled. There was no significant difference (*P* > 0.05) between males (mean ± SD, 381 ± 385 repeats, range 70–2500) and females (mean ± SD, 326 ± 141 repeats, range 105–570). 

### 3.3. Normal [CTG] Distribution

**Figure 2:**
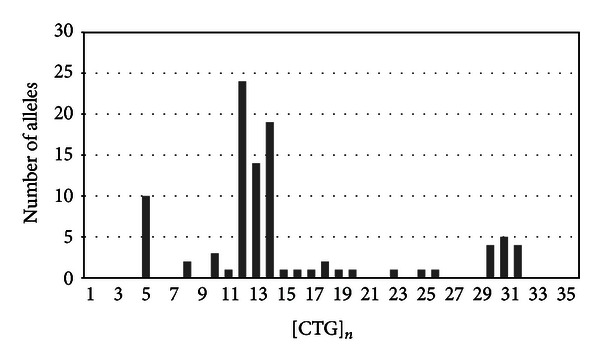
Distribution of [CTG] expansions in 96 normal alleles.

Instead, the histogram presented in Figure 2 describes the distribution of nonexpanded normal [CTG] repeats length in 96 alleles from 48 normal subjects. The preponderance of normal individuals was heterozygous; only five patients presented homozygosity with five [CTG] repeats. After capillary electrophoresis, we observed nineteen different types of alleles and the size of [CTG] repeats ranged from 5 to 32. The allele most frequently observed presented 12 [CTG] expansions (24/96; 25%), followed by 13 (14/96; 14.6%), and finally by 14 (19/96; 19.8%) repeats. Thirteen patients presented the large normal alleles between 30–32 repeats (13/96; 13.5%), and none of the subjects had numbers of repeats near cut-off area (38 < [CTG] < 50). 

### 3.4. Pitfalls of Molecular Analysis

**Figure 3:**
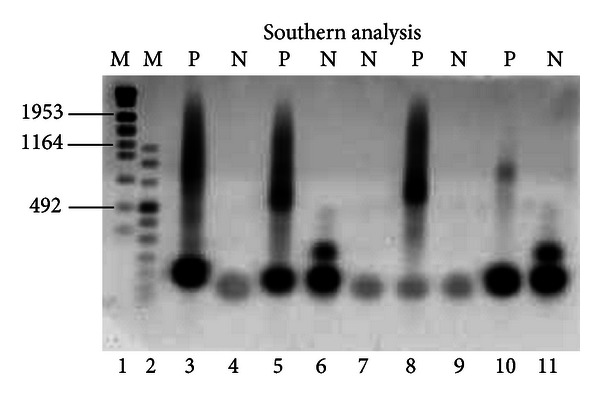
Expansion detection by Southern Blot Analysis. Lanes 1 and 2 are DNA Molecular Weight Marker, (VII and VIII, resp., Roche Diagnostics). Results for DM1 affected individuals are shown in lanes 3, 5, 8, and 10. Results for DM1 unaffected individuals are shown in lanes 4, 6, 7, 9, and 11, but three of these are homozygous normal (lanes 4, 7, and 9), confirmed by TP-PCR.

Our experience demonstrates that, although conventional Long-PCR associated to Southern Blot Analysis proves to be accurate enough to detect large DM1 expansions, it is unsuitable for the identification of premutated or protomutated alleles and alleles with small [CTG] size.  Figure 3 shows three of the five homozygous-normal alleles (lines n° 4, 7, and 9) that our assay detects with a single band, while usually the heterozygous-normal alleles occur in two distinct bands (lines n° 6 and 11). As suggested by the guidelines, we went further with the analysis and confirmed these homozygous normal patients by TP-PCR: all homozygous patients analyzed were healthy.

### 3.5. Cardiac and Neuromuscular Assessments

To see whether [CTG] size correlated to muscle and cardiac impairment muscle strength, myotonia and ECG abnormalities were correlated to expansion size. Myotonia was the most frequent symptom at onset (about 70%), followed by distal muscle weakness (56%). There was no correlation between symptom at onset and [CTG] size. One quarter of our patients had abnormal ECGs (PR intervals > 200 msec; QRS duration > 120 msec) but had no symptoms suggestive of cardiac involvement. [CTG] expansion size did not correlate to the presence or absence of ECG abnormalities or degree of muscle weakness as assessed by MRC values. 

## 4. Discussion

Myotonic Dystrophy type 1 belongs to a group of repeat disorders where an aberrant expansion of normally short tandem repeats in specific genes, known as “dynamic mutations,” causes the disease. Molecular analysis represents, in these types of diseases, an essential tool to confirm the symptomatic manifestations, but it is also a predictive test. Currently, Southern Blotting of genomic DNA, digested with an appropriate restriction enzyme, has been the gold standard for the detection of DMPK alleles, with the use of several different probes for hybridization [27]. However, this procedure has a small false-negative rate because of the reduced sensitivity in cases of extreme somatic heterogeneity. For this reason, a Long-PCR associated with Southern Blot Analysis is still widely used and recommended for this type of disease. In this study, we developed and validated a molecular diagnostic method Myotonic Dystrophy SB kit, based on this type of methodology. The strong point of this assay is that all reagents are pre-packaged and ready to use. The analytical results, evaluated on a total of 113 DNA samples, in terms of sensitivity, specificity and accuracy were very high (>99%), and both prospective and retrospective analysis gave no false positives or false negatives. The opportunity, for molecular biology laboratories, to have CE-IVD marked product available, greatly reduces the probability of failures during PCR amplification or Southern Blot Analysis. This may reach 10% in some cases. On the other hand, the limitations related to the identification of premutated alleles and alleles with small [CTG] size can be overcome by checking the homozygous normal patients by TP-PCR. TP-PCR, for DM1detection, represents a robust and reliable PCR method that can rapidly identify the presence of expanded alleles for any disorder caused by repeat expansions. Although it can distinguish between healthy homozygous and affected heterozygous samples with no length restriction, it is not able to determine the exact size of the repeats over a certain threshold, that is, it is very important to allow correlation studies. The association of two molecular methods as a Long-PCR and Southern transfer, together with Triplet-repeat Primed (TP)-PCR [28, 29], is strongly recommended because they should be able to detect a wide range of mutations. 

The analysis of distribution of large normal alleles can help to study the prevalence of DM1 in northern Italy. The different frequency of alleles with more than 20 [CTG] repeats also depends on ethnic groups [30] belonging and are not a pathologic cause in the individual but have been considered as a danger in the successive generation. In our study, the retrospective analysis showed that the most frequent normal allele presented 12 [CTG] repeats, and no individual carried a premutation allele. While the predominant pathologic [CTG] expansion size, in our population, was in the “*E2a*” range. The fact that no correlation was found between expansion size and muscle strength and ECG abnormalities should not be considered a limitation of molecular analysis and thus of the method, but it should be interpreted in the light of the instability of the expansion size and of tissue mosaicism. Where possible, correlations should be made between size of [CTG] expansion of the tissue involved and symptoms related to that tissue or system. In conclusion 20 years have passed since the [CTG]*n* repeat expansion mutation was discovered in patients with Myotonic Dystrophy type 1. Although much has been learned within this period, an identification and characterization of a biomolecular DM1 testing, such as that described in this paper, could be very helpful in clinical practice. 

## Figures and Tables

**Table 1:** Analytical performances of Myotonic Dystrophy SB kit compared to different “homemade” assays.

Total *n* = 113	*Myotonic Dystrophy SB kit		“Homemade” assays	
	DM1^+^	DM1^−^	DM1^+^	DM1^−^
DM1-positive (*n* = 65)	65	0	65	0
DM1/DM2-negative (*n* = 32)	0	48	0	48
DM1-negative/DM2-positive (*n* = 13)	0	13	0	13

*Sensitivity = >99%, specificity = >99%.

**Table 2:** Distribution of [CTG] expansions in 65 DM1 patients.

Molecular diagnosis	Clinical phenotype	[CTG] repeats	Number of patients
Premutation		38–50	0

DM1	“Mild”	51–149	9
	“Classic”	*E2a* 150–450	42
		*E2b* 451–2000	12
	“Congenital”	>2000	2
		Total DM1 patients	**65**
